# Joining forces to understand what matters most: qualitative insights into the patient experience of outpatient rheumatology care

**DOI:** 10.1093/rap/rkad068

**Published:** 2023-08-16

**Authors:** Madeleine J Bryant, Rebecca Munt, Rachel J Black, Amy Reynolds, Catherine L Hill

**Affiliations:** Rheumatology Unit, The Queen Elizabeth Hospital, Central Adelaide Local Health Network,, Woodville South, SA, Australia; Rheumatology Unit, Royal Adelaide Hospital, Central Adelaide Local Health Network, Adelaide, SA, Australia; School of Medicine, Faculty of Health Sciences, University of Adelaide, Adelaide, SA, Australia; Nursing Education, Central Adelaide Local Health Network, Adelaide, SA, Australia; Adelaide Nursing School, Faculty of Health Sciences, University of Adelaide, Adelaide, SA, Australia; Rheumatology Unit, The Queen Elizabeth Hospital, Central Adelaide Local Health Network,, Woodville South, SA, Australia; Rheumatology Unit, Royal Adelaide Hospital, Central Adelaide Local Health Network, Adelaide, SA, Australia; School of Medicine, Faculty of Health Sciences, University of Adelaide, Adelaide, SA, Australia; Patient Research Partner; Flinders Health and Medical Research Institute (Sleep Health), Flinders University, Bedford Park, SA, Australia; Rheumatology Unit, The Queen Elizabeth Hospital, Central Adelaide Local Health Network,, Woodville South, SA, Australia; Rheumatology Unit, Royal Adelaide Hospital, Central Adelaide Local Health Network, Adelaide, SA, Australia; School of Medicine, Faculty of Health Sciences, University of Adelaide, Adelaide, SA, Australia

**Keywords:** patient experience, care quality, patient-centred care, qualitative research

## Abstract

**Objective:**

People with rheumatic diseases are frequent, long-term attenders of health-care services. Their care experiences are central to improving services. The aim of this study was to explore real-world experiences and priorities of people attending outpatient rheumatology care and those of health-care professionals (HCPs) providing care.

**Methods:**

This qualitative study consisted of five semi-structured focus groups. Participants included rheumatology outpatients (*n* = 16) of two tertiary teaching hospitals and HCPs (*n* = 14; rheumatologists, rheumatology trainees, physiotherapists, a specialty nurse and a pharmacist). Participants explored priorities when attending outpatient services, real experiences and aspirations for improving future care. Transcripts were coded using inductive and deductive thematic analysis.

**Results:**

Seven key themes were identified: smooth flow of technical processes, care coordination, individualized care, information sharing, clinical excellence, patient empowerment and comprehensive care. The findings were aligned conceptually with quality standards in Australia and worldwide. Different sub-themes and prioritization of concerns emerged from patient and HCP subgroups. Highly prioritized themes for patients pertained to processes and technical aspects of care. HCPs focused on themes relating to non-technical aspects of service provision: information sharing, individualization of care, patient advocacy and empowerment.

**Conclusion:**

This study captured valuable insights into the current experience of outpatient rheumatology care from the perspective of patients and HCPs. It informs a collective understanding of differing and shared priorities, positives of current care and areas requiring change. Themes derived from the study data can be conceptualized in terms of the process, content and impact of care. Such domains can be measured longitudinally by routine implementation of validated patient-reported experience measures in rheumatology.

Key messages

- Research prioritizing the experiences of patients has an important place, alongside quality care indicator and outcome measure data, and can drive change in how rheumatology care is delivered.
- Our study suggests that rheumatology patients and health-care professionals place high importance on receiving and providing care that is individualized and patient centred.
- Logistics and care processes can pose barriers to the provision of high-quality care and have a negative impact on patients.
- There is great potential to capture experience-related data from patients routinely and use this directly to reduce such barriers and improve patient services.

## Introduction

Health-care systems continue to evolve as the end of the first quarter of the 21st century approaches. Medicine and health care are no longer delivered in a traditionally didactic way, in which patients assume a passive, receiving role [1, 2]. There is growing awareness that patients are expert consumers in matters pertaining to their health and have extensive experiential knowledge beyond consultation-level issues of individual autonomy and management decisions.

Leading bodies, including the World Health Organization (WHO), recognize that people-centred health services can deliver more efficient, cost-effective and equitable care by consciously seeking the perspectives of individuals and communities when designing interventions [3]. Globally, health-care systems are changing to implement routine and widespread partnering with patients: during direct care, through greater consultation and through mutual leadership of system and policy design and implementation [4–6]. A systematic review examining optimal engagement of patients in service design, delivery and evaluation concluded that engagement yields numerous positive outcomes at the macro level, including patient-centric policy development, enhanced governance and increased health education [1]. At an institutional level, engaging patients can promote collaboration and mutual learning by changing the culture of staff and care settings [1]. For individual patients, benefits of enhanced partnership are manifold; they include greater empowerment and independence and enhanced quality of life [7].

A key foundation underpinning partnership in direct care provision is information gathering about care experiences, coupled with interventions to promote shared responsibility [2]. To this end, prior work in a rheumatology context has highlighted the need for purpose-built instruments to routinely collect and integrate patient experience-related data into quality assurance systems [8]. People with rheumatic diseases engage with health services on a frequent and ongoing basis; in many cases, this interaction will be lifelong [9–13]. Their experiences and opinions of care are therefore central to improving care quality [14], and research prioritizing the experiences of patients has an important place alongside other care indicator and outcome measure data, both broadly and in the specialty of rheumatology. Patient-reported experience measures (PREMs) designed to reliably capture patient experiences will probably become a routine component of quality and safety reviews in health-care settings. Early development of PREMs has occurred in rheumatology as a specialty; however, further validation of specific instruments is required to ensure their relevance to the lived experience of patients [8].

The aims of this study were as follows: to capture the current experiences of care in outpatient rheumatology services, from both patient and health-care professional (HCP) perspectives; to explore patient views on what is considered ideal patient-centred care; to explore HCP views on what constitutes high quality delivery of patient-centred care; and to explore similarities and differences of opinion between cohorts.

## Methods

### Study design

This non-interventional study was designed in accordance with published guidance on qualitative methodology [15, 16], using an interpretive phenomenological approach to examine the lived experiences of participants represented in the sample and to understand commonalities and differences in priorities and concerns [17–20]. Focus groups in qualitative research are a means of facilitating engagement with multiple participants in a time-effective way, while addressing broad research questions and yielding a blend of perspectives from the interaction between individual participants [21, 22].

### Patient and public involvement

Our patient research partner contributed to qualitative analysis and manuscript preparation. Study findings will be disseminated via Arthritis Australia [23].

### Sample and recruitment

#### Patients

**Figure 1.**
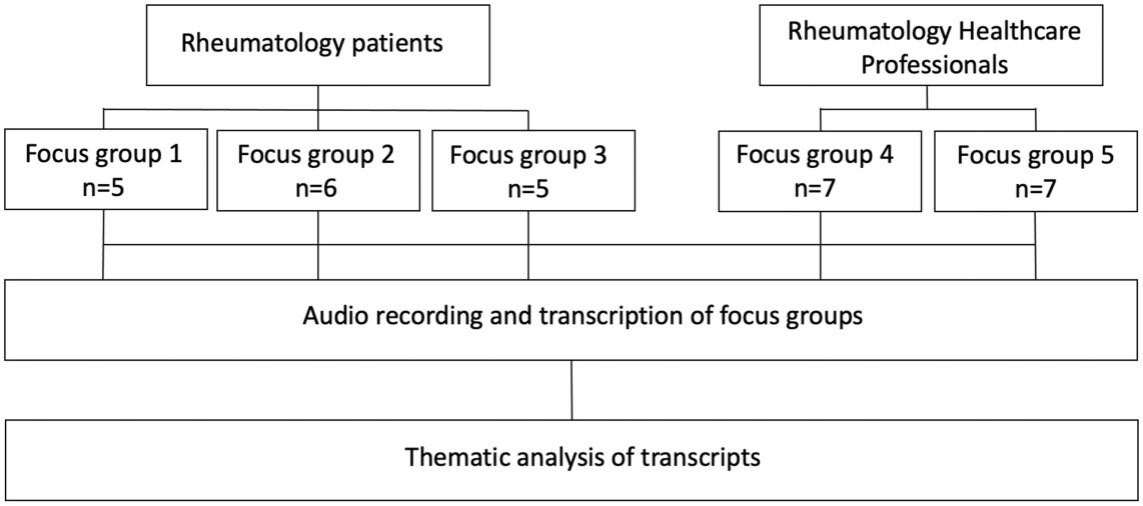
Study design

Individuals attending outpatient rheumatology clinics at two Australian tertiary hospitals (The Queen Elizabeth and Royal Adelaide Hospitals) were invited to participate in semi-structured focus groups (Fig. 1). Potential participants were identified in clinic by the rheumatology nurse or doctor, or from existing patient cohort databases. Purposive sampling of cases was used to achieve *a priori* targets of biological sex, age, cultural background, disease diagnosis and duration. Groups were assembled with representation of demographic factors in each. Eligibility criteria included age >18 years, ability to communicate in English, and attendance at outpatient clinics within 12 months at either recruitment site. Patient focus groups were conducted at both hospital sites, in November and December 2021. Transport vouchers were provided.

#### Health-care professionals

Two focus groups comprising rheumatology HCPs were conducted, including medical, specialty rheumatology nursing, pharmacy or physiotherapy providers within public or private rheumatology clinics. Invitations to participate were sent by email to rheumatology staffing groups across the recruitment sites and to specialist rheumatologists working in the private sector. HCP focus groups were conducted in December 2021 by virtual format owing to coronavirus disease 2019 restrictions.

The study was approved by the Central Adelaide Local Health Network Human Ethics Committee (reference 13846).

### Data collection

**Figure 2.**
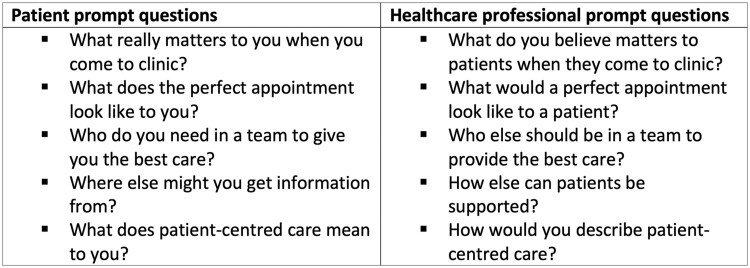
Standardized prompt questions employed in focus groups

Written informed consent was obtained, including consent for audio recording and transcription. Patient focus groups were facilitated by an experienced qualitative researcher (R.M.) and observed by an additional investigator (M.J.B.). HCP focus groups were facilitated by two investigators (M.J.B. and C.L.H. or R.J.B.). Participants in all groups were invited to discuss perspectives on their care priorities when attending outpatient services (patient groups) and providing care (HCP groups). This included personal experiences of clinic attendance, opinions on patient-centred care and aspirations for future improvement. Groups followed a semi-structured format, with standardized prompt questions developed by the authors (Fig. 2) to promote discussion around care domains that could be addressed by a PREM, such as how care occurs, care content and impact on individuals. The question guide was informed by prior work on rheumatology patient experiences in published literature [24, 25].

Focus groups were audio-recorded, transcribed verbatim using a professional transcription service, and imported for coding into NVivo v.12 software (QSR International).

### Analysis

Focus group transcripts were analysed using both inductive and deductive approaches, using six phases of thematic analysis as described in published texts on qualitative methodology [19, 20]. Open coding was performed independently by three coders (M.J.B., C.L.H. and R.M.) [18]. Conceptual links between individual perspectives were explored both within and between the patient and clinician groups. Analysis occurred in a recursive manner, with ongoing recruitment until data saturation and concept repetition were achieved [15]. A working thematic framework was identified throughout the analysis period [20]. Potential key themes were identified as those repeatedly raised and explored in depth during the focus groups [20]. The research team collectively reached consensus on the themes to be reported.

## Results

### Participant characteristics

Forty-three patients and 15 clinicians were invited to participate. Five semi-structured focus groups were conducted with 30 participants: three patient groups (total *n* = 16 in groups of *n* = 5, *n* = 5 and *n* = 6) and two HCP groups (total *n* = 14 in two groups, each of *n* = 7). Focus group sizes followed qualitative research guidance on recommended sample size and dynamics [26]. Reasons for non-participation included unsuitable scheduling (patients: *n* = 4; HCP: *n* = 1) or not interested in study objectives (patient *n* = 7).

Patient participant characteristics are summarized in Table 1. Participant age ranged from 29 to 79 years, with a median (interquartile range) age of 63 (14.5) years. Fifty per cent of participants were female. The median (interquartile range) disease duration was 9 (19) years. A range of cultural backgrounds, education level and employment states were represented. RA was the most frequent primary rheumatological diagnosis (*n* = 8).

**Table 1.** Summary demographic data for patient focus group participants

Patient characteristics	
Sex, female, %	50
Age, median (interquartile range), years	63 (14.5)
Disease duration, median (interquartile range), years	9 (19)
Primary rheumatological diagnosis, *n*	RA, 8 PsA, 3 AS, 3 Vasculitis, 1 Gout, 1
Self-reported ethnicity, *n*	Australian, 9 Aboriginal Torres Strait Islander, 2 British, 1 Italian, 1 Polish, 1 Filipino, 1 Not specified, 1
Language background, *n*	English, 13 English as second language, 3
Education, highest level, *n*	Primary schooling, 3 Secondary schooling, 3 Trade/apprenticeship, 3 Certificate/diploma, 4 Bachelor degree or higher, 3
Employment status, *n*	Full time, 1 Unemployed, 3 Retired, 8 Disability pension, 2 Home duties, 1 Not specified, 1

### Health-care professionals

Represented in the sample were specialist rheumatologists (*n* = 8), rheumatology trainees/registrars (*n* = 2), physiotherapy (*n* = 2), specialty nursing (*n* = 1) and pharmacy (*n* = 1). Sixty-four per cent of participants were female. The median (interquartile range) time in practice was 9.5 (15) years. Eight participants worked solely in public rheumatology clinics, two participants worked in both public and private clinics, and four worked solely in private clinics. One participant reported English as their second language. The predominant cultural background reported was Australian (*n* = 12); others included Sri Lankan (*n* = 1) and Australian Chinese (*n* = 1).

### Themes

**Figure 3.**
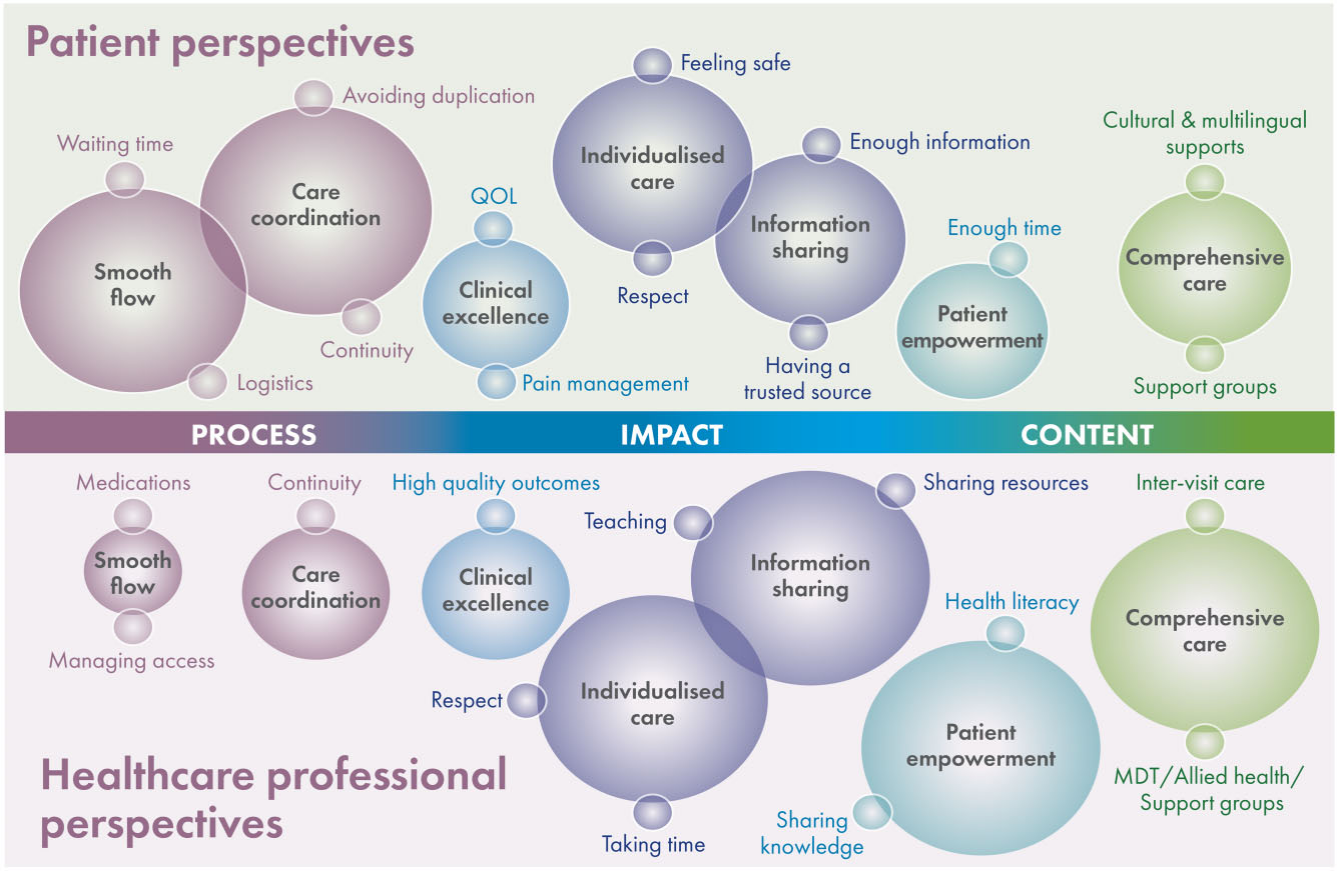
Conceptual model of findings

Seven key themes were identified, summarized in Tables 2 and 3 and Fig. 3. Although themes were shared across patient and HCP cohorts, different sub-themes and prioritization of concerns emerged from these distinct groups. Highly prioritized themes for patients pertained to the processes and technical aspects of care (waiting times, appointment flexibility and continuity of care), whereas HCPs focused on themes relating to non-technical aspects of service provision, such as information sharing, individualization of care, patient advocacy and empowerment.

**Table 2.** Themes and subthemes from qualitative data analysis of patient focus groups

Theme	Subtheme	Patient voice
Smooth flow of care processes	Technical clinic processes (e.g. waiting time) Appointment logistics (e.g. booking, scheduling) Inter-visit care (e.g. flare management) Managing results	‘An hour later, you're still waiting there.… I've got more important things to do.’ C, age 63 years ‘Waiting is what you do. It’s why I always bring a book.’ G, age 50 years ‘My time is as important as the doctor's time.’ J, age 70 years
Care coordination	Continuity of care Seeing the same clinician Knowing who is responsible Issues with repeating ones’ story at each appointment	‘I don't believe I've ever seen the same doctor twice.’ B, age 78 years ‘I repeat the same thing over and over and over again.’ C, age 63 years ‘(when) the doctor has already read your notes … it’s really good.’ P, age 72 years
Individualized care	Sense of feeling heard and respected Sense of feeling safe Receiving care that is patient centred	‘As long as I’m listened to.’ G, age 50 years ‘I guess the one thing we have in common is we’re all trying to be heard.’ G, age 50 years
Information sharing	Receiving information about my condition Receiving information about my medications Being able to ask questions about symptoms or side effects	‘Really helpful to have … some sort of print out of resources that might be available.’ D, age 62 years ‘Giving us … a handout at your first appointments.’ H, age 47 years ‘I come in here expecting to know more (about) my condition.’ E, age 54 years ‘It's important that we do seek out information.’ D, age 78 years
Clinical excellence	Commitment to high-quality disease outcomes Disease control Adequate pain management Communication about results	‘I'm grateful that after trialling all this stuff, I'm on an injection now and I can walk.’ C, age 63 years ‘If you've had a level two pain for 300 days in a row, it starts to affect you.’ H, age 47 years ‘If they expect you to do the blood test, at least just give me the basic information about the blood test.’ C, age 63 years ‘I get blood tests every 3 months. I'd like to be told about it.’ C, age 63 years
Patient empowerment	Clinicians taking time to explain Clinicians taking time to answer questions Being involved in medication decisions	‘The rheumatologist just saying to you look, we think this has been happening … or we think everything is stable.’ D, age 62 years ‘(I) always like to know the reports.’ B, age 29 years
Comprehensive care	Accessing patient support groups Access to interpreters Access to Aboriginal Health Workers Multilingual resources Feeling able to bring family or support person	‘Speaking with people who do have the same condition can be ever so helpful.’ D, age 78 years ‘(In online groups) people just talk about what’s going on and what treatments they’ve got, what medications.’ B, age 29 years ‘I've not seen an Aboriginal worker there at all … over 10 years.’ C, age 63 years Online support groups can be ‘really handy … a whole group of people with all their different experiences that you can call on.’ H, age 47 years

**Table 3.** Themes and subthemes from qualitative data analysis of health professional focus groups

Theme	Subtheme	Health-care professional voice
Comprehensive care	Involvement of allied health professionals Working as part of a multidisciplinary team Referring to patient support groups and organizations Inter-visit care (e.g. flare plans) High-quality documentation	‘Drawing in all of the team if it's a multidisciplinary approach to a disease.’ L, Rheumatology nurse ‘Certainly. a dietitian, an exercise physiologist, hand therapy, OT [are essential to a team].’ L, Rheumatology nurse ‘In that diary there are pointers in regards to identifying a flare and answering those questions and then going on to implementing the flare plan.’ L, Rheumatology nurse ‘Patients really value knowing when they leave the room … how to get back in touch, or how, if things go wrong between that 3 months ‘til they’re due to see us again.’ C, Rheumatologist
Information sharing	Providing condition information Developing and sharing resources Radiology and results as teaching aide	‘Using aids such as their own radiology images.’ S1, Rheumatology trainee ‘We use aids such as information that's written in the correct language.’ J1, Rheumatologist ‘There's a … (web)site which has videos for people who may not be able to read.’ P, Rheumatologist ‘Make sure they go home with some information to read.’ P, Rheumatologist
Individualized care	Providing care that is patient centred Adapting communication style Creativity in helping patients to understand Seeing patient as more than diagnosis Taking time to understand context behind referral	[Patient-centred care involves] ‘everyone involved in the patient's care … the patient themselves and their family too.’ S2, Rheumatologist [Patient-centred care means] ‘being unhurried.’ J2, Rheumatologist ‘Listening to the patient and seeing why they've actually come.’ L, Rheumatology nurse ‘Informing them as best as I can, but then respecting that it's their choice.’ S, Physiotherapist ‘Some patients really want their family members to be involved in their care as well … being open to that.’ S3, Rheumatologist ‘Seeing that the clinician appreciates you as more than just your kind of problem that you're there for … sees you as a person.’ J1, Rheumatologist
Patient empowerment	Awareness of health literacy Sharing knowledge about condition Naming and explaining medications Facilitating patient self-advocacy Facilitating sense of control Patient as information owner	Patients valued ‘clarity around what they were meant to be taking and what changes are to be made.’ M, pharmacist ‘It's a clinician's job to then tailor your explanation (according to health literacy).’ S4, Rheumatologist ‘From a medication perspective, knowing how to get more scripts or how to access more medications if needed.’ M, pharmacist ‘Involving the patient in the decision-making.’ S1, Rheumatology trainee ‘There’s lots of things that we can actually engage the patient (with), and I always try and frame it that together we can … we’re far more powerful together if we engage.’ L, Rheumatology nurse
Care coordination	Continuity of care Seeing the same clinician, or knowing who is responsible	Understanding that patients ‘don't want to have to explain the whole situation over again.’ J3, Rheumatologist ‘They like to have very clear lines of communication that they can access easily as well.’ T, Rheumatology trainee
Clinical excellence	Commitment to high-quality disease management	‘Something that (patients) want is that you have … expertise in the condition.’ J3, Rheumatologist ‘If I come across a difficult situation and I have to hit the literature, I make a note … something that can be read by the patient or by the GP.’ P, Rheumatologist
Smooth flow of care processes	Managing access to clinicians Managing medication access Differences between public and private clinics	‘The experience at the front desk I think is really important … the fact that you have staff that care.’ S3, Rheumatologist ‘Patients in the private setting do feel a little bit more empowered, because they've made that choice to come and see you. It would be nice if the public sector patients also had a similar feeling.’ J1, Rheumatologist ‘Administration staff are part of that really crucial team.’ S3, Rheumatologist ‘People who come into private probably have a slightly higher expectation for … being contactable and being available between visits.’ K, Rheumatologist ‘The service available and the resources and care available, I don't think it's that different between public and private.’ K, Rheumatologist

GP: general practitioner; OT: occupational therapy.

### Patient perspectives

#### Smooth flow of care processes

This theme was raised extensively by patients during focus groups, encompassing many technical clinic processes and logistics, including pre-appointment and on-the-day factors, managing results and between-visit care. Participants repeatedly discussed the negative impact of long waiting times on their experience of care, although it was accepted by many as an inherent feature of the health-care system. Waiting time was raised as an issue throughout all patient groups and was interwoven with many different aspects of their clinic experience, with conceptual links to other themes, such as feeling respected or otherwise, and having enough time with a HCP as opposed to feeling rushed.

#### Care coordination

Issues of care coordination and care continuity were cited recurrently by patients as key priorities. Specific examples included seeing the same HCP at each visit, not having to provide clinical details repeatedly, and the importance of HCPs knowing and acknowledging individual history and context, a concept closely related to feeling valued.

#### Individualized care

Notions of feeling heard and respected and of having a sense of safety were central to definitions of patient-centred care among the patient cohort.

#### Information sharing

Patients frequently cited the importance of being able to access information about their condition and treatment from a trusted source. A specific example included the need for opportunities to ask questions and learn about medications. This fed into definitions of high-quality care. The role of the internet and support groups for information provision was a further recurring subtheme.

#### Clinical excellence

From the patient perspective, this theme encompassed concepts such as good disease control and symptom management, in addition to a need for HCPs to be aware of the impact of symptom burden and disability on quality of life.

#### Patient empowerment

This theme, conceptually related to that of self-advocacy, was explored frequently by patients through a logistical or process-based lens, for example in relationship to having sufficient or insufficient time with a HCP to have their concerns addressed.

#### Comprehensive care

Patients were vocal in describing lack of access to support groups and self-management programmes, in addition to gaps in access to multilingual and culturally inclusive resources.

### Health-care professional perspectives

#### Comprehensive care

This theme was the most deeply discussed theme among HCP participants, encompassing issues such as multidisciplinary team involvement, patient organizations, cultural awareness and sensitivity, and involvement of family and support people.

#### Information sharing

Among HCPs, this theme manifested in discussion about the high importance placed on sharing and explaining information, including finding creative ways to facilitate patients learning from their own health data.

#### Individualized care

Health-care professionals explored this concept in terms of what practical adaptations in care provision are made to account for individual factors and health literacy. HCPs articulated the importance of acknowledging context and of identifying and addressing the specific concerns of the individual as essential to delivering ideal care.

#### Patient empowerment

For HCPs, this was a highly prioritized and explored theme. This cohort discussed in detail the need to be aware of and adjust for individuals’ health literacy, adopting a shared approach to decision-making and knowledge-sharing and ensuring ownership of individual health data by the patient.

#### Care coordination

This theme was explored in terms of theoretical ways to optimize efficiencies of care, by avoiding repetition and duplication of processes. HCPs were highly aware of the negative impact of this domain on the patient care experience.

#### Clinical excellence

Among the HCP cohort, this theme manifested in discussion around commitment to achieving high-quality clinical outcomes, encompassing subthemes, such as satisfactory disease control, and issues of pain management, disability and access to medications.

#### Smooth flow of care processes

Among HCPs, technical and process-based subthemes were not as frequently discussed as other themes. Examples of HCP views on this theme included the differences between public and private clinics in relationship to inter-visit access to clinicians and medications.

## Discussion

This study provides insights into the key concerns and priorities of rheumatology patients and health-care providers from two tertiary hospitals and private practice in Australia. The dominant themes are conceptually aligned with domains of care represented within quality standards in Australia and worldwide [27–29]. In particular, principles from the WHO global strategy on people-centred health services and the Australian Charter of Healthcare Rights (ACHR) resonate with themes derived from these data [3, 30]. The ACHR describes essential principles underpinning standards of care, including domains of respect, partnership and information, access and safety [30]. Participants from both patient and HCP cohorts in this study highlighted the importance of having ‘respect for each other’ (patient C, age 63 years) and as clinicians, ‘respecting (patients’) decisions’ (S, physiotherapist), demonstrating respect as a fundamental and mutually accepted cornerstone of patient-centred care. The pursuit of partnership was likewise demonstrated as a key aspiration from both cohorts, with patients identifying the role of the therapeutic relationship or ‘share(d) history.. (as) extremely important’ (patient P, age 70 years) and HCPs expressing awareness that individuals and care teams are ‘far more powerful together if we engage’ (L, Rheumatology nurse). Furthermore, an emphasis on empowering individuals to ‘connect in with community services’ (A, physiotherapist) was also evident, a theme which, importantly, aligns with the WHO strategic direction on Empowerment, Engagement and Co-Production, and enabling individuals to take control of their own health needs [3].

In this study, we have been reminded of the importance placed by patients on receiving adequate information about their condition and treatment and of the essential role that this plays in fostering patient engagement and empowerment. Patients described the ‘really helpful’ role of supporting resources (patient D, age 62 years), in addition to the desire for continued learning: the ‘expect(ation) to know more (about) my condition’ (patient E, age 54 years). This conceptual emphasis was reflected by the HCP cohort describing many ways in which information sharing can be adapted and individualized by using illustrative patient results, pictorial resources and multilingual material. That these themes are familiar at both a macroscopic and direct-care level suggest that they remain as authentically relevant to the individuals in the present sample as they are to systems and governance structures on a much larger scale [18, 31].

Overall, we have identified that patient and HCP cohorts are concerned with the same issues, although prioritization is different between groups. Several disparities were noteworthy; the foremost of which was a discord in appreciation of the impact on logistics on the overall care experience. Issues of waiting time and appointment inflexibility were demonstrated as universal patient concerns, and their negative impact on care was grossly evident in patient cohort data. Patients indicated that logistics and processes can significantly impede provision of high-quality clinical care; clinicians cited awareness of such factors, although less cogniscence of their scale, a mismatch with potential to disconnect the entire patient–clinician interaction. Patients reported sentiments such as ‘waiting is what you do’ (patient G, age 50 years), yet one has ‘more important things to do’ (patient C, age 63 years), views demonstrating the negative impact of logistics on sentiments such as feeling respected or engaged in partnership. HCPs might well be aware of system-level inefficiencies and logistical frustrations, but these are rarely visible to the individual patient experiencing real-time delays or cancellations. Research in other contexts has demonstrated the importance of proactively informing patients of delays in order to mitigate the negative psychological impact of long outpatient waiting times [32]. To this end, system-level solutions are needed to improve the patient experience of logistics: measures such as attention to staffing levels and clinic flow and adopting technology to keep patients informed are likely to enhance sentiments of autonomy and respect within the patient-clinician encounter.

Also notable for the contrast between patient and HCP cohorts within this study was the differing emphasis placed on non-technical aspects of care; in particular, the expression and exploration of themes such as empowerment and comprehensive care. Among the patient cohort, empowerment was largely explored in terms of being involved in decision-making and having sufficient time to have concerns addressed. HCPs described many different manifestations of empowerment, such as the importance of facilitating patient self-advocacy, acknowledging patient ownership of health information and adjusting care to account for individual health literacy. This theme was explored in detail by HCPs and was given high priority in descriptions of high-quality care but was not as strongly evident in patient focus group data. Likewise, the theme of comprehensive care revealed a divide between patient and HCP cohorts. For patients, this theme encompassed preferences for greater access to formal support groups, self-management tools and culturally sensitive resources, including interpreting services. In contrast, HCPs focused on the importance of factors such as involving the allied health and multi-disciplinary team and attention to inter-visit care. HCPs explored in depth the need to provide care that is both patient centred and individualized, including seeing patients as more than a diagnosis, understanding biopsychosocial context and seeking creative ways to engage and partner. All of this commentary demonstrated optimism and aspiration in how patient-centred care can be delivered. Despite this, a disparity between cohorts in the terms and language used to describe these phenomena suggests that patients are not yet sufficiently enabled to seek out such important non-technical elements of care.

When describing ‘patient-centred care’, patients cited the way an encounter made them feel: heard, respected or seen, as examples. Meanwhile, HCPs described in more practical, literal terms the elements of routine practice they believed to represent individualization of care: being unhurried, involving support persons and facilitating individual treatment decisions based on tailored information and advice. Clinicians readily cited multiple examples of patient-centred care at junctions within a consultation; yet signposting these to patients in real time might not be one of them, as evident in the finding that patients did not describe this experience. Bridging this divide will require clinicians as a profession to incorporate increased and regular patient feedback on non-technical care domains, review blind spots in care provision and adapt practice accordingly.

This study was conducted in a metropolitan setting, thus limiting generalizability of findings to the rural and remote patient experience. Also, although this study did include HCPs from the private setting, patients attending private rheumatology clinics were not represented. Within the dual-sector model of Australian health care, the public system provides free access to hospital health-care services, whereas self-funded private health cover affords choice of provider and location [33]. In this study, we endeavoured to represent a cohort with comparatively less control over access to care by focusing on experience in the public system. Future representation of patients in the private sector and rural settings might provide useful comparison, in addition to individuals with multi-system autoimmune diseases, such as SLE, inflammatory myopathy and SSc, whose characteristics and experiences of care might vary from those identified in this work. Despite these limitations, we believe the findings have relevance to outpatient rheumatology settings beyond the constructs of the study, owing to the diversity of the patients and HCPs represented in the sample.

This study used purposive sampling, wherein prospective participants are identified as qualified to address the study aims, which is an approach frequently adopted in qualitative methodology to select information-rich cases with experience of the question of interest [34], albeit one that confers potential bias. The study followed convention in focus group methodology by continuing to recruit participants until theoretical saturation of concepts was achieved, suggesting a comprehensive understanding of participant concerns. Rheumatologists were the most represented practitioner type, which might have contributed to accentuation of the views of this subgroup, but which reflects real-world service structure in the region of study, where patients attending public rheumatology care see a rheumatologist (or trainee) far more frequently than other allied health professionals.

Rheumatology patients typically attend outpatient services every 3 or 6 months, with a large cohort requiring more frequent appointments, inter-visit follow-up and investigations. This study has demonstrated the significant overlap between process-based and non-technical domains of care within a rheumatology context, such as the burden of waiting times and inflexible processes, and the downstream impact on domains such as feeling respected, achieving high-quality clinical standards and efficient resource allocation. Collecting and analysing experiential data for this cohort is essential to providing care that better addresses patient needs and priorities. Themes derived from this study can be conceptualized in terms of process, content and impact of care, which are domains that can be captured through routine implementation of PREMs [35–38]. Routine collection of PREM data has been linked to service improvement and broad behavioural change [39], in addition to enhancements in patient engagement and empowerment [40–42]. Improving engagement between individuals, communities and health-care providers can achieve goals of better-quality care [2] and efficiency and equity in uptake of service [3], making it an urgent priority in modern health-care delivery.

### Conclusion

This study has captured valuable insights into the current experience of outpatient rheumatology care from the perspective of patients and HCPs. It informs a collective understanding of differing and shared priorities, positives of current care and areas requiring improvement. Building on knowledge, future work will seek to further the role of purpose-designed and validated PREMs in routine rheumatology care, as an adjunct to other quality and outcome measure instruments.

## Data Availability

The data underlying this article will be shared on reasonable request to the corresponding author.
